# Supplementary material for: SCoPE-MS: mass spectrometry of single mammalian cells quantifies proteome heterogeneity during cell differentiation
Source: Genome Biol. 2018 Oct 22;19:161. doi: 10.1186/s13059-018-1547-5 (PMC6196420; doi:10.1186/s13059-018-1547-5)
Supplement: Supplementary file 1 — Figure S1. Contribution of background noise to quantification of peptides in single cells. Figure S2. Relative quantification is independent from the carrier channel. Figure S3. Accuracy of SCoPE-MS quantification. Figure S4. Confidence of peptide identification and its effect on quantification. (PDF 805 kb) [file 13059_2018_1547_MOESM1_ESM.pdf]

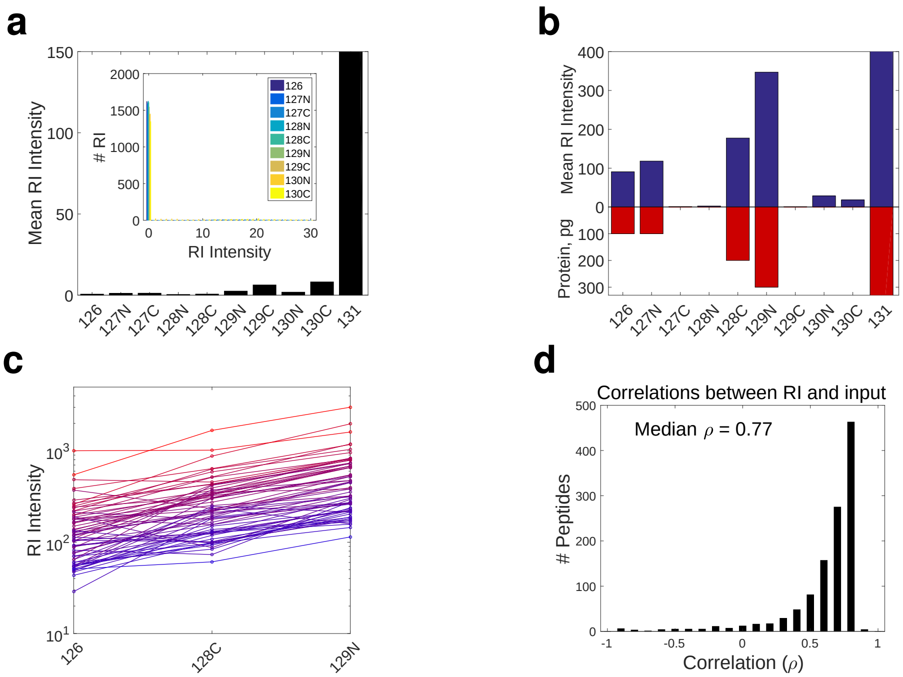

Fig S1 | Contribution of background noise to quantification of peptides in single cells. (**a**) Reporter ion (RI) intensities in a SCoPE set in which the single cells were omitted while all other steps were carried out, i.e., trypsin digestion, TMT labeling and addition of carrier cells in channel 131. Thus, RI intensities in channels $126-130C$ correspond to background noise. The distribution of RI intensities in the inset shows that the RI for most peptides in channels $126-130C$ are zero, i.e., below the MaxQuant noise threshold. The y-axis is limited to 150 to make the mean RI intensities visible. The mean RI intensity for single-cell channels is about 500. (**b**) Mean RI intensities for a TMT set in which only 6 channels contained labeled proteome digests and the other 4 were left empty. Channels 126, 127N, 128C, and 129N correspond to peptides diluted to levels corresponding to 100, 100, 200 and 300 picograms of cellular proteome, channel 131 corresponds to the carrier cells (bars truncated by axes), and the remaining channels were left empty. The RI for most peptides are not detected in the empty channels, and their mean levels very low. This suggests that background noise is low compared to the signal from peptides corresponding to a single cell. (**c**) The intensities for representative RIs from (b) are plotted, color coded by their mean intensity. The results show that both lowly (blue) and highly (red) abundant RIs exhibit the expected scaling with the increase of the labeled cell lysate. (**d**) For more representative display of the scaling of each peptide with the input amount, the correlation between the sample input amount and the RI intensities for each peptide are computed and the distribution of correlations for all peptides displayed.

a

Experimental Design Table

Label	Set 1	Set 2	Set 3
126	Jurkat	Jurkat	Jurkat
127N	U-937	U-937	U-937
127C	Jurkat	Jurkat	Jurkat
128N	U-937	U-937	U-937
128C	Jurkat	Jurkat	Jurkat
129N	U-937	U-937	U-937
129C	Jurkat	Jurkat	Jurkat
130N	*empty*	*empty*	*empty*
130C	U-937	U-937	U-937
131 Carriers	200 Jurkat	200 U-937	200 HEK-293

b

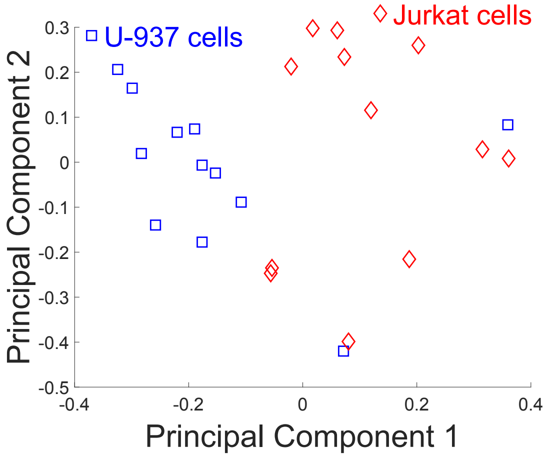

c

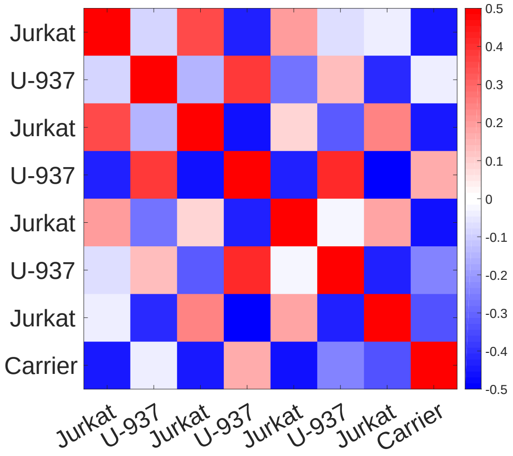

Fig S2 | Relative quantification is independent from the carrier channel. (**a**) Design of control experiments used to test the ability of SCoPE-MS to distinguish U-937 cells from Jurkat cells independently from the cells in the carrier channel. The carrier channel in each SCoPE-MS set contained 200 cells: 200 Jurkat cells in set 1, 200 U-937 cells in set 2, and 200 HEK-293 cells in set 3. (**b**) Unsupervised principal component (PC) analysis using data for quantified proteins from the experiments described in panel (a) stratifies the proteomes of single cancer cells by cell type regardless of the type of cells used in the carrier channel. (**c**) To explore the extent to which peptides from the carrier channel might affect relative quantification in the single-cell channels, we computed the pairwise correlations between the relative peptide levels of a SCoPE-MS set. Relative peptide levels were computed by first normalizing the RI intensities in each channel to a median of 1 (to correct for different amount of total protein, especially in the carrier channel) and then by dividing the vector of RI intensities for each peptide by its mean, to remove the large differences in abundances between different peptides. The correlations between these relative estimates indicate that Jurkat cells correlate positively to Jurkat cells and negatively to U-937 cells. The converse holds for U-937 cells. Importantly, the carrier channel correlates negatively with most single cells, except for a weak correlation with one U-937 cell, perhaps reflecting slightly high contribution of U-937 cells to the carrier channel. (**d**) We computed the correlation between the sample input amount and the RI intensities for each peptide and plotted the distribution of correlations for all peptides

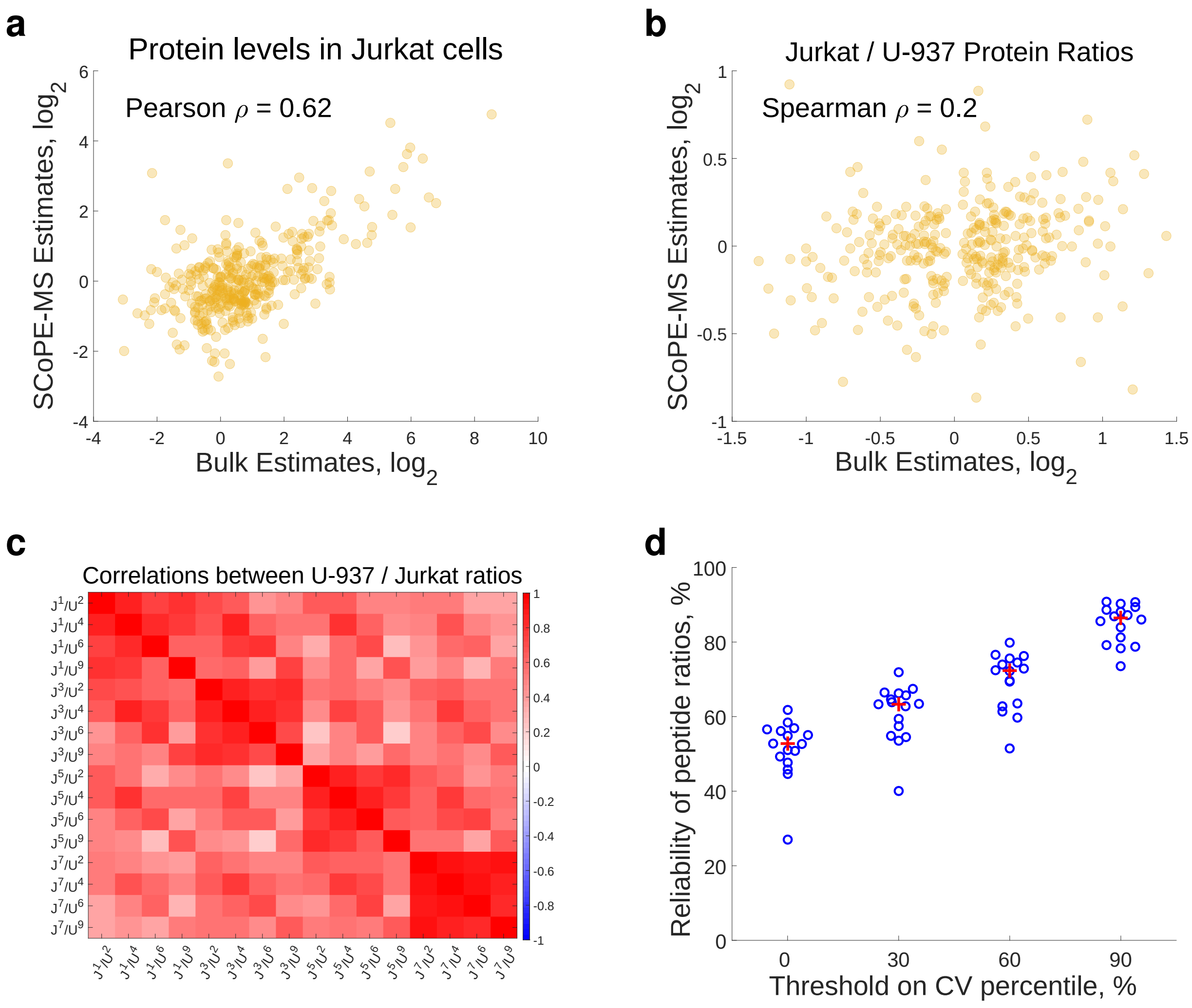

Fig S3 | Accuracy of SCoPE-MS quantification. (**a**) Comparison between protein levels estimates from bulk samples and from single cells. The single-cell protein estimates are the average from 12 Jurkat cells from the experiments described in Fig. 1b and equal as the summed up precursor ion areas apportioned by RI intensities. (**b**) Comparison between relative protein levels (fold changes) estimated from bulk samples and from single cells. The single-cell protein estimates are the ratio between average levels summed across 12 Jurkat cells over the average level summed across 12 U-937 cells. Proteins whose bulk estimates did not change between Jurkat and U-937 cells (fold change less than 10%) were omitted from the plot. (**c**) A correlation matrix of all pairwise Pearson correlations among the ratios of peptide abundances in U-937 and in Jurkat cells from Set 2 in Fig. 1b. The superscripts corresponds to the TMT labels ordered by mass, with 1 being 126, 2 being 127N and so on. The positive correlations among estimates from different combinations of TMT channels suggest good consistency of relative quantification. (**d**) Distributions of correlations between technical replicates of peptide ratios measured in two halves of the same single-cell set; each measurement estimated the peptide ratios from peptides corresponding to 1/2 cell. The first distribution correspond to correlations from across all measured peptides. The other distributions correspond to the correlations computed from the subset of peptides having coefficient of variation (CV) above the indicated percentile, i.e., peptides with larger fold changes. The red crosses mark the distribution medians. Correlations were computed with log transformed protein levels and ratios.

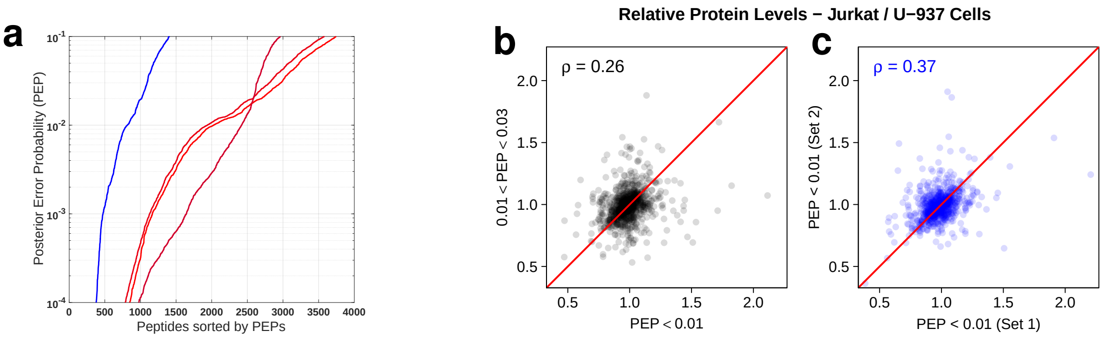

Fig S4 | Confidence of peptide identification and its effect on quantification (**a**) The peptides from a few SCoPE-MS sets from Fig. 1 and from Fig. S3 were rank sorted by the confidence of their identification as quantified by the posterior error probability (PEP). The rank sorted PEPs for each set are color-coded based on the number of quantified peptides at PEP < 0.03, excluding peptides from contaminant proteins. The results exemplify the variability in the number of peptides quantified in different SCoPE-MS sets. For some sets, relaxing the false discovery rate (FDR) to 3% increases significantly the number of quantified peptides while keeping false positives below 3%. (**b**) Protein ratios derived from peptides having PEP $\in (0.01,\ 0.03]$ correlate positively with the corresponding protein ratios derived from peptides having PEP < 0.01, thus indicating that peptides identified with lower confidence carry quantitative information. (**c**) Protein ratios derived from two non-overlapping subsets of peptides having $PEP < 0.01$. The correlation for peptides identified with lower confidence (panel b) is lower than for those identified with high confidence (panel c). This might be due in part to the fact that factors reducing the confidence of identification, such as lower abundance or higher co-isolation, are also likely to affect quantification.
